# Pico-Watt Scanning Thermal Microscopy for Thermal Energy Transport Investigation in Atomic Materials

**DOI:** 10.3390/nano12091479

**Published:** 2022-04-27

**Authors:** Seunghoe Koo, Jaehee Park, Kyeongtae Kim

**Affiliations:** Department of Mechanical Engineering, Incheon National University, Incheon 22012, Korea; shkoo7736@gmail.com (S.K.); pekyk2959@gmail.com (J.P.)

**Keywords:** scanning thermal microscopy (SThM), pico-watt, nano scale heat transfer, atomic scale heat transfer, ultra-precision, highly ordered pyrolytic graphite (HOPG)

## Abstract

The thermophysical properties at the nanoscale are key characteristics that determine the operation of nanoscale devices. Additionally, it is important to measure and verify the thermal transfer characteristics with a few nanometer or atomic-scale resolutions, as the nanomaterial research field has expanded with respect to the development of molecular and atomic-scale devices. Scanning thermal microscopy (SThM) is a well-known method for measuring the thermal transfer phenomena with the highest spatial resolution. However, considering the rapid development of atomic materials, the development of an ultra-sensitive SThM for measuring pico-watt (pW) level heat transfer is essential. In this study, to measure molecular- and atomic-scale phenomena, a pico-watt scanning thermal microscopy (pW-SThM) equipped with a calorimeter capable of measuring heat at the pW level was developed. The heat resolution of the pW-SThM was verified through an evaluation experiment, and it was confirmed that the temperature of the metal line heater sample could be quantitatively measured by using the pW-SThM. Finally, we demonstrated that pW-SThM detects ultra-small differences of local heat transfer that may arise due to differences in van der Waals interactions between the graphene sheets in highly ordered pyrolytic graphite. The pW-SThM probe is expected to significantly contribute to the discovery of new heat and energy transfer phenomena in nanodevices and two-dimensional materials that have been inaccessible through experiments.

## 1. Introduction

The rapid advancement of material science and nanotechnology [1] has led to the development of various atomic materials, such as nanoparticles (zero-dimensional (0D)) [2], nanowires (1D) [3,4], 2D atomic materials [5,6], and thin films deposited using atomic layer deposition (ALD) [7,8,9]. Owing to the development of various atomic materials, innovative applications, such as molecular-scale transistor devices, high-efficiency photovoltaic devices, high-efficiency energy storage batteries, and bio-molecular devices, have broadened the scope of research in various fields [10,11,12].

To realize innovative technologies using atomic materials, understanding the structural properties of crystals and the characteristics of energy carriers (electrons, phonons, and photons) that transmit and convert energy as well as reveal novel energy transformation phenomena in the ultralocal region, is essential [13,14,15]. Therefore, to develop new atomic materials, it is necessary to investigate and verify the energy transfer and conversion phenomena of energy carriers at atomic and nanoscale levels. According to the second law of thermodynamics and the principles of quantum mechanics, energy loss is inevitable during energy transfer and conversion, thereby resulting in thermal phenomena. In other words, it is possible to investigate and characterize the transmission and conversion characteristics of energy carriers by measuring thermal phenomena.

Measurement systems that can investigate energy transfer and conversion phenomena at the nano or atomic scale are very limited. Currently, time-domain thermal reflectance (TDTR) [16,17], Raman spectroscopy [18,19,20], and scanning probe microscopy (SPM) techniques [21,22] are used to investigate in extremely limited conditions, and local thermal phenomena are not effectively measured. Therefore, a precise measurement method that is capable of approaching both spatial and signal characteristics is required. Scanning thermal microscopy (SThM), which has the highest spatial resolution, is the most promising candidate for thermal measurements at the atomic scale [23,24,25]. The concept of SThM was conceived in the early 1990s, and it has been continuously developing since then. The highest performing SThM reported so far is the ultra-high vacuum (UHV) SThM with a thermal resolution of 300 pW developed by Kim et al., combining with the UHV environment [26]. It has made a significant contribution to the field of nanoscale heat transfer phenomena with extraordinary measurement results for the transfer of radiative heat energy in the ultra-close range of ~2 nm, heat transfer through a single molecular channel, and heat dissipation effect by the graphene thin film [27,28,29,30]. However, most of these studies are limited to experiments using point measurements. The SThM have been considered a successful technique for obtaining an insight into thermal energy transport phenomena regarding to its applicability at the nano scale. However, futher progress is required to achieve more precise temperature resolution and spatial resolution as the size of samples shrink [31,32]. Especially, heat flow through the single molecule or atomic junction is resulting low thermal conductance values at tens of pico-watts per kelvin [29]. The experimental limitation of detecting such small conductance remains unrealized. Considering the recent rapid development of atomic materials, it is necessary to develop an SThM that is ~10 times more sensitive and capable of precise scanning to measure the thermal properties according to the molecular and crystal structure of the material.

In this study, efforts were made to develop a pico-watt SThM (pW-SThM) equipped with an ultra-precision calorimeter to measure and elucidate energy transfer and conversion phenomena at nano and atomic scales. Additionally, it was verified that the pW-SThM could measure ~16 pW of heat change through various experiments. Finally, in order to demonstrate the applicability of the developed pW-SThM to atomic materials, it was used to observe the tiny difference of the thermal conductance in a highly ordered pyrolytic graphite (HOPG) sample, which is an ideal 2D material.

## 2. Materials and Methods

### 2.1. Microelectromechanical System (MEMS) Process for Probe

**Figure 1:**
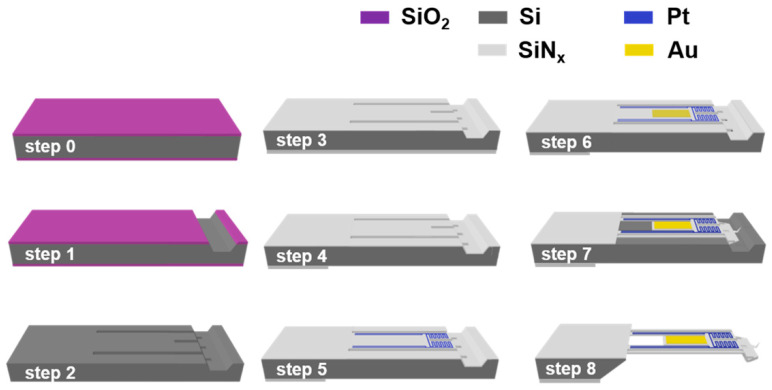
Schematic of the MEMS process for probe fabrication.

The fabrication process of the probe is described as follows (Figure 1). (Step 0) The fabrication process starts by thermally growing ~1 μm-thick silicon oxide film on a silicon wafer. (Step 1) The silicon oxide film is patterned via plasma etching, and the exposed Si is etched using KOH to create a trapezoidal groove. (Step 2) The remaining silicon dioxide film is etched via buffered oxide etching, and the wafer is patterned using plasma etching to create a U-shaped cantilever. (Step 3) Subsequently, a 500 nm thick silicon nitride (SiN_x_) film was deposited using low pressure chemical vapor deposition (LPCVD) on both sides of the wafer. (Step 4) To facilitate the release process using KOH etching in the final step, the rear side of the wafer is patterned via plasma etching. (Step 5) Next, a Pt line pattern (Cr/Pt: 5/50 nm) is defined via the lift-off process to fabricate a calorimeter that precisely measures the amount of heat. (Step 6) Subsequently, a Au line (Cr/Au: 5/90 nm) is lithographically defined via a lift-off process. (Step 7) The front side is lithographically patterned through plasma etching, and then the tip of the probe is patterned via plasma etching more than twice. (Step 8) Finally, the SThM probe is released via KOH etching. The tip of the probe is manufactured using a silicon etching process (anisotropic etching) instead of a deposition process.

### 2.2. Evaluation Experiment for Thermal Resolution of Probe

A current was supplied to the probe using current source (6221), and the voltage signal generated from the probe was measured using a lock-in amplifier (SR830). A small current of amplitude (*I_f_* = 10 μA) is maintained and the frequency-normalized temperature graph is investigated for frequencies from 0.5 Hz to 50 Hz. A frequency at 2 Hz is kept and the applied power-temperature increase graph is investigated for a temperature increase from 0 K to 14 K. We obtained the electrical resistance of probe and temperature coefficient of resistance in the same way as reported in the previous study [26]. The four-probe resistance is measured to obtain the electrical resistance of the probe. The time constant and thermal resistance of the probe were obtained from the frequency-normalized temperature graph and the applied power-temperature increase graph, respectively. The measured time constant is 36 ms. It relates to the scan rate required for reaction time during scans of the sample in Section 2.3 and Section 2.4. Subsequently, measuring the power spectrum density (PSD) noise voltage using an FFT spectrum analyzer (SR760), the temperature resolution of SThM was obtained. Additionally, the temperature increase with respect to the applied heat using the lock-in amplifier, the minimum temperature change that the probe could detect was obtained. The frequencies *f* = 0.5, 2, and 20 Hz were used.

### 2.3. Quantitatitve Temperature Profile of the Metal Heater Line Sample

The quantitative temperature measurement is performed using atomic force microscopy (AFM) (RHK Beetle ATM 350) and a developed probe. A current (*I* = 10 mA) at a frequency of *f* = 5 Hz is applied to the Pt line heater. The temperature of the metal increases at ~20 K above the ambient temperature (the temperature of the sample by obtaining from the 3ω method is detailed in the Supporting Information Figure S1). The thermal sensor of the probe is calibrated through the temperature rise obtained by contacting the probe to the sample so that its temperature is known, as in previous study [26]. The temperature image was obtained by monitoring for voltage generated from the probe while the topography was measured using the AFM contact mode with the resolution of 128 × 128 pixels. Considering the time constant in Section 2.2, the sample was scanned with scan speed was 2 μm/s. The contact force between the probe and the sample is kept constant 90 ± 3 nN. A line profile is obtained by averaging of 128 pairs of lines for the measured area. The result represents the displacement-temperature curve from the center of the metal heater, and is compared with the theoretical value obtained through the finite element method (FEM). All experiments were performed by maintaining a pressure of ~5 × 10^−6^ Torr in the high vacuum.

### 2.4. Measurements of Thermal Properties of HOPG through SThM

Thermal properties of HOPG is measured using AFM and a developed probe. The topography and temperature images are obtained simultaneously by scanning the HOPG, maintaining the temperature of the probe at a temperature of ~120 K higher than the ambient temperature (~293 K) and the contact force of 50 ± 3 nN between the probe and the sample. Topography and temperature images in the 600 nm × 600 nm and the 300 nm × 300 nm regions were measured using AFM contact mode with a resolution of 128 × 128 pixels and 256 × 256 pixels at a scan rate of 40 nm/s and 20 nm/s, respectively (as in Section 2.3, the scan speed was determined by considering the time constant in Section 2.2).

## 3. Results and Discussion

### 3.1. Composition and Principle of pW-SThM

**Figure 2:**
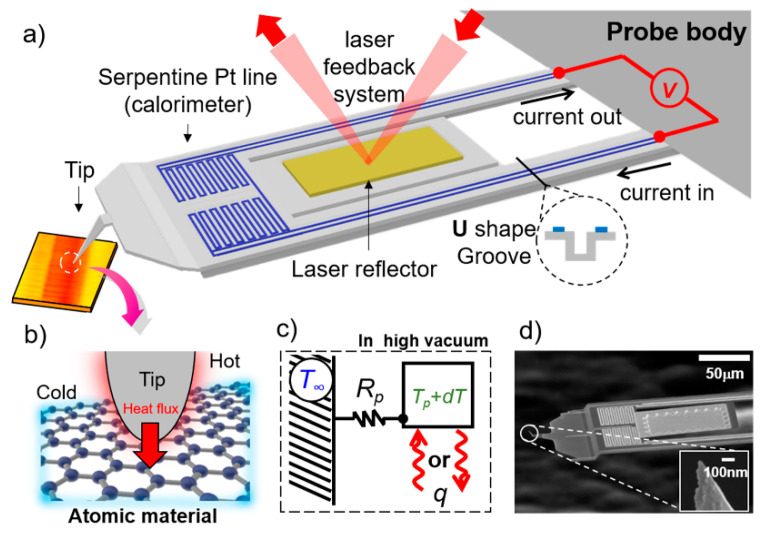
(**a**) Schematic illustration of the pW-SThM. Calorimeter composed of a serpentine platinum line is present in the probe. Temperature of the probe is measured by monitoring the electrical resistance of the metal line. (**b**) Schematic diagram of heat transfer between the probe and substrate. When the tip (at the end of probe) and the substrate make contact, heat transfer occurs through nano-scale channels. (**c**) Schematic of the temperature change of the probe due to change in its thermal resistance caused by heat flux in high vacuum. *T_∞_*, *T_p_*, d*T*, *q*, and *R_p_* are the room temperature, temperature of the SThM probe, temperature change, heat flux, and thermal resistance of the SThM probe, respectively. (**d**) Scanning electron microscopy (SEM) images of the fabricated SThM probe. The tip of the probe is very sharp with a diameter less than ~100 nm.

Figure 2a shows a schematic of the pW-SThM system developed in this study. The probe is composed of cantilevers with sufficient mechanical stiffness, a laser reflector, a calorimeter made of a serpentine Pt line, and a tip protruding at the end of the probe. In the SThM system, as shown in Figure 2b, heat transfer occurs through a nano-scale contact formed between the probe and sample when the probe contacts the sample. Therefore, temperature and thermal properties can be measured with the highest spatial resolution. Sensing the extremely small heat flux transferred to or escaping from the probe through a calorimeter determines the heat resolution of the probe. Figure 2c shows the principle of the thermal response of the probe from the outside. The heat flux transferred to the probe, as shown in Figure 2c, can be expressed as follows:

(1) $$Q=\frac{dT}{R_{p}}$$

where d*T* is the temperature change of probe, and *R_p_* is the thermal resistance of the probe. The heat resolution as the probe is able to measure can be expressed *Q_res_* = *T_res_*/*R_p_* from the Equation (1), where *T_res_* is the limiting temperature resolution of probe. Considering the equations, the realization of a sensitive probe that can detect even a small temperature change and a high probe thermal resistance is a significant strategy to increase the heat resolution. However, owing to the characteristics of the probe, the mechanical properties (stiffness of the cantilever) should also be considered because it is mounted on an atomic force microscope.

### 3.2. Pico-Watt Thermal Resolution Probe Design and Fabrication

In this study, to achieve the pW thermal resolution of the probe, a high-resolution calorimeter and probe cantilever were designed to ensure high thermal and mechanical performance, respectively. Sadat et al. manufactured a calorimeter with a high resolution of ~μK using a platinum line film and performed noise analysis [33]. Herein, a calorimeter with a temperature resolution of ~μK that could be inserted into the probe was designed. Sufficient mechanical stiffness is an important factor for maintaining a stable contact of the probe, and to obtain a high thermal resolution of the probe, the design of the cantilever with high thermal resistance is crucial. If the cantilever is thin and long, the thermal resistance increases; however, it is mechanically unstable. Conversely, a thick and short cantilever is mechanically stable but causes a decrease in the heat resolution because of the low thermal resistance of the probe. To fabricate a probe with a high thermal resistance (~10^6^ K/W), the cantilever has been designed with a sufficient length of ~200 μm and a very thin silicon nitride thin film of ~500 nm. Additionally, a U-shaped groove has been inserted to increase the mechanical stability. The spring constant of the probe manufactured using this design was ~1.86 N/m, which was determined using the FEM. This is detailed in Supporting Information Figure S2. In conclusion, the probe was designed to measure tens of pico-watts and heat transfer at the atomic scale through components with a high temperature resolution and thermal resistance. The designed probe was manufactured through an MEMS process, and hundreds of probes were manufactured simultaneously in one batch. Figure 2d shows the SEM image of the probe fabricated in this study.

### 3.3. Time Constants and Thermal Resistance of the Probe

**Figure 3:**
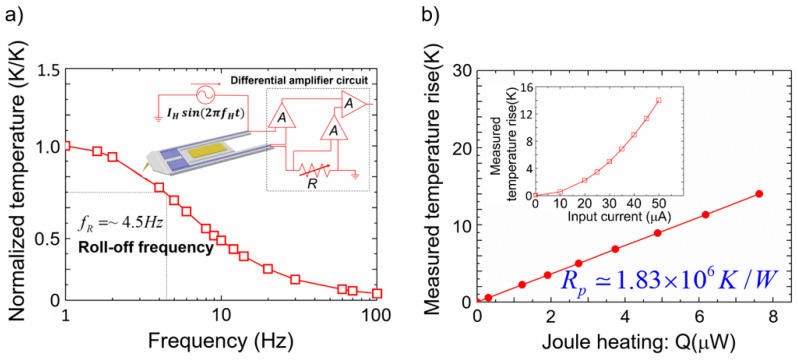
(**a**) Normalized measured temperature of the probe with varying frequency of the applied heat. Thermal response of the probe decreases as the frequency increases in accordance with the thermal time constant of the probe. Inset shows the schematic diagram of circuit for measuring the time constant and thermal resistance of the probe. Probe is heated through an AC power supply, and the normalized temperature is measured. Signal from the probe is amplified and detected using the amplifier circuit for precise measurement, and A and R are the amplifier and resistance, respectively. (**b**) Measured temperature rise according to the power applied to the probe (frequency of the applied voltage is fixed at 2 Hz.) Slope of the graph gives the thermal resistance of the probe (*R_p_*). Inset shows the input current-temperature graph.

The performance of the manufactured probe was verified through various experiments. To investigate the response of the probe to the frequency and input power, an amplifier circuit was fabricated, as shown in Figure 3a. When the modulated current flows, an electrical signal related to modulation is measured, and the measured temperature rise is defined as follows [33]: *T* = *V*_2*f*_/[*I*_1*f*_*·R*_0_*·α*], where *V*_2*f*_ is the voltage to be measured; *I*_1*f*_ is the input current; *R*_0_ is the electrical resistance of probe at room temperature, and *α* is the temperature coefficient of resistance. The values of the electrical resistance of probe and temperature coefficient of resistance were used 3052 ± 1 Ω and 0.0019 K^−1^, respectively. If the frequency is gradually increased while the voltage applied to the probe is kept constant, the magnitude of the temperature reduces according to the response of the probe. The results of the measurement of the normalized temperature with respect to frequency are shown in Figure 3a. The thermal time constant of the probe is defined as *τ* = (2*·π·f_R_*)^−1^, where *f_R_* is the roll-off frequency, defined as the frequency with a temperature value of $1/\sqrt{2}$ K/K in the normalized temperature graph. The thermal time constant *τ* = ~36 ms of the probe is calculated using *f_R_* = 4.5 Hz obtained from the graph in Figure 3a. Next, if the applied voltage is gradually increased while maintaining the frequency at 2 Hz, the temperature increases linearly with the applied power, owing to the thermal resistance of the probe (Figure 3b). Using Equation (1) and the results shown in Figure 3b, the thermal resistance of the probe has been estimated to be ~1.83 × 10^6^ K/W. All measurements have been recorded inside a high vacuum system of ~10^−6^ Torr.

### 3.4. Temperature Resolution and Heat Resolution of the Probe

**Figure 4:**
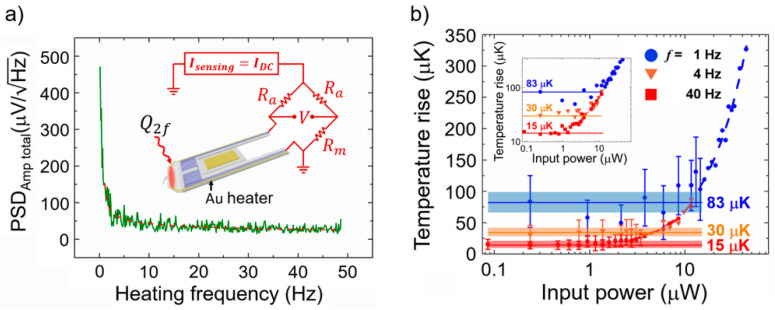
(**a**) Measured PSD of thermal noise in the calorimeter of the SThM probe. Inset shows the Wheatstone bridge circuit used for temperature resolution measurement. *R_a_* and *R_m_* are the resistance and matching resistance, respectively. (**b**) Measured temperature rise of the probe using a lock-in amplifier, when the heater line is excited using sinusoidal heating currents (0.5, 2.0, and 20 Hz). Solid lines show the estimated noise floor. Dashed line represents a trend line of temperature. Error bars reflect the standard deviation in the RMS value measured by the lock-in amplifier. Shaded areas represent the noise region where the temperature cannot be measured. Inset presents the same data in a log-log plot. The temperature of the probe is proportional to the input power in the temperature range beyond the temperature resolution.

The temperature resolution of the probe was estimated by measuring the PSD of the voltage noise and performing an experiment using a lock-in amplifier. Sadat et al. theoretically analyzed the noises (Johnson noise, shot noise, 1/*f* noise, etc.) generated when the calorimeter is operated and estimated the temperature resolution by measuring the voltage noise [33]. The inset in Figure 4a shows the experimental schematic used in this study for measuring the temperature resolution. A circuit was fabricated using a measuring probe, matching resistance, and adjustable resistance, and an experiment was conducted in which heat could be applied to the probe using an Au line heater. For precise temperature measurements, the heating signal was an AC signal (*f* ≠ 0) with a specific frequency, and the probe sensing signal was a DC signal (*f* = 0) [33]. The PSD measurement results are shown in Figure 4a, and the temperature resolution according to the frequency was obtained through *T_res_* = (*PSD_Amptotal_*/[*I·R*_0_*·α*]) × *bandwidth*. In this experiment, a bandwidth of 12 mHz was used, and when the frequency of the applied heat signal was 4 Hz, the temperature resolution of the probe was ~29 μK. Additionally, for comparison with the PSD measurement results, the temperature resolution was obtained by using a lock-in amplifier. When voltages with various frequencies (1*f*) were applied to the gold heater mounted on the probe, temperature oscillations (2*f*) occurred. The temperature variation value that the probe could measure was investigated while applying a power of various amplitudes corresponding to the frequencies of 0.5, 2, and 20 Hz. The temperature resolution was measured at frequencies of 1, 4, and 40 Hz (*bandwidth* = 12.5 mHz), and the results are shown in Figure 4b. The probe does not detect a temperature change at a temperature lower than the temperature resolution, whereas it increases linearly with respect to the input power at a temperature higher than the temperature resolution (inset in Figure 4b). The measured temperature resolutions are ~83, ~30, and ~15 μK for frequencies of 1, 4, and 40 Hz, respectively, and these results match well with the PSD measurement results. In conclusion, the probe had a higher temperature resolution than ~30 μK at frequencies above 4 Hz. Considering the thermal resistance and temperature resolution obtained from the experimental results, the heat resolution of the probe obtained using Equation (1) was ~16 pW. Consequently, a probe was developed through a complex MEMS process that can measure the heat transfer at a level of ~16 pW, as verified experimentally.

Additionally, an indigenously developed mirror circuit was applied in the experiments. The mirror circuit was devised to effectively eliminate the drift noise. When measuring the temperature using a DC signal, temperature signal drift occurs according to the change in the outside temperature. Matching probes manufactured via the same MEMS process were installed in a vacuum chamber, and a perfectly symmetrical circuit was constructed (Supporting Information Figure S3). Both probes were heated to the same temperature and resulted in the same drift in response to ambient temperature. The output voltage *V_out_* was determined through the voltage differences (*V_p_*–*V_a_* and *V_m_*–*V_b_*) of the probes and resistors in the circuit, such as *V_out_* = (*V_p_*–*V_a_*) − (*V_m_*–*V_b_*), where *V_p_*, *V_a_*, *V_m_*, and *V_b_* are the voltage outputs from the sensing probe, resistor *R_a_*, matching probe, and resistor *R_b_*, respectively. As a result, by subtracting the same drift value from each other, the effect of the ambient temperature drift could be effectively eliminated.

### 3.5. Quantitative Temperature Profile of the Metal Heater Line Sample

**Figure 5:**
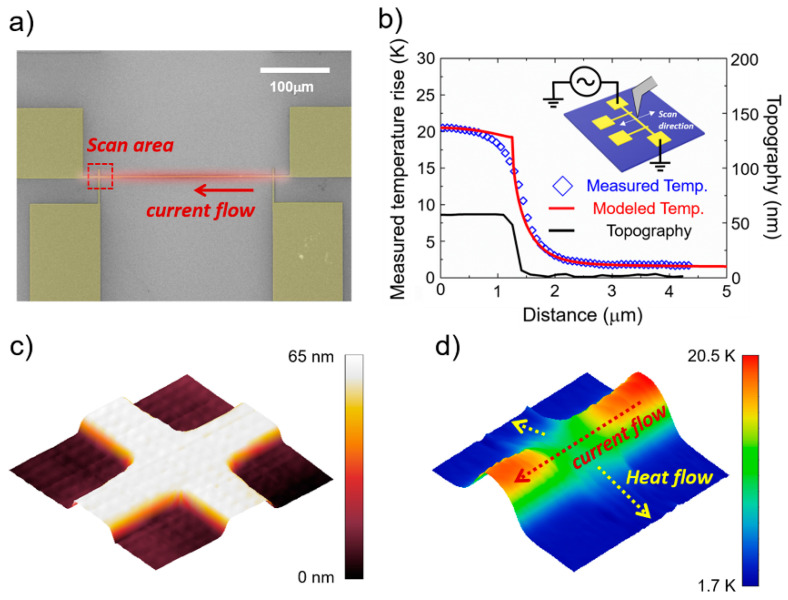
(**a**) SEM image of the fabricated platinum line heater sample. When an electric current is applied to a metal heater, a temperature field is generated around the metal line. (**b**) Graph of the topography and temperature distribution of the metal heater sample according to the distance from the center of the metal line obtained by applying a uniform sinusoidal current to the heater. Black line, blue diamond, and red line represent topography, experimentally measured temperature distribution, and simulated temperature distribution, respectively. Inset shows schematic for the measurement of the temperature distribution of a metal heater. (**c**) Three-dimensional graph of topography obtained by measuring the area marked in (**a**) (10 μm × 10 μm). (**d**) Three-dimensional graph of the temperature distribution in the area same as (**c**). Temperature is high on the line through which current flows owing to Joule heating, but on the cross area where two lines cross, the temperature is low because heat escapes to the other line.

To prove that the developed probe could perform a quantitative temperature scan, the temperature profile of a metal heater sample was studied. The sample was fabricated as a platinum line film with a line width of ~2.5 μm and a thickness of 55 nm (Cr/Pt: 5 nm/50 nm—Cr is adhesion layer) on 0.5 μm thick silicon dioxide, as shown in Figure 5a. The inset of Figure 5b shows a schematic of the temperature measurement setup. When a current is applied to the sample, the temperature increases owing to Joule heating, and this temperature increase and distribution can be experimentally verified through the 3ω method [34]. The results of the 3ω experiment for examining the temperature of the metal line heater are given in Supporting Information Figure S1. The topography and temperature images were recorded simultaneously while scanning the metal line area. The results are shown in Figure 5b by averaging the line profile in the direction away from the metal center point. Additionally, to confirm the validity of the measured temperature, the results of the theoretical analysis using the heat transfer equation (red line) and the experimental results (blue scatter) obtained by scanning have been compared. The calculations of the theoretical temperature distributions are provided in Supporting Information Figure S4. As the distance from the metal line increases, the temperature rapidly decreases, and the temperature profile obtained experimentally matches the theoretical result. From this result, it can be confirmed that quantitative temperature measurement is performed well.

For nanoscale heat transfer measurement, the intersection of the two metal lines was scanned. The topography and temperature distribution of the area where the two metal lines cross (red dashed rectangle in Figure 5a) are shown in Figure 5c,d, respectively. The topography image depicts the same height and shape of the lines with the same thickness. However, an evident temperature difference can be observed in the temperature distribution. This is because one line generates heat owing to the current flow while the other line acts as a heat sink. Through the measured thermal image, it was demonstrated that precise local measurement was realized for the Joule heating and heat transfer phenomenon generated by the crossed two metal lines.

### 3.6. Measurement for Thermal Conductance of HOPG

**Figure 6:**
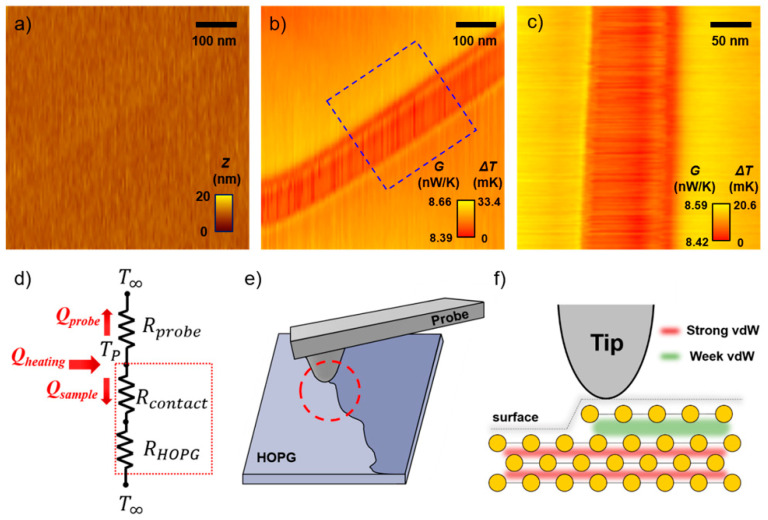
(**a**) Topography and (**b**) temperature images obtained simultaneously from a thermal scan of an HOPG sample (600 nm × 600 nm). (**c**) Enlarged image of the region marked by the blue rectangle in (**b**) (300 nm × 300 nm). (**d**) Thermal resistance network defined from the resistance to heat flow in the sample and SThM probe. *R_probe_*, *R_contact_*, and *R_HOPG_* are the thermal resistance of probe, interfacial thermal resistance, and thermal resistance of the HOPG, respectively. (**e**) Schematic for the measurement of the HOPG sample. Graphene sheet on the surface of the HOPG may be cracked or torn out between the sheets. (**f**) Schematic showing the difference in van der Waals (vdW) forces between graphene sheets.

In order to show that heat transfer and thermal conductance of atomic materials can be measured very precisely at the nanoscale, an HOPG sample was prepared and thermal images were measured. The results are shown in Figure 6. HOPG is an ideal atomic material that consists only of carbon atoms. Additionally, because graphene sheets have high mechanical stiffness and thermal conductivity, they have been used in various studies, such as in research on structures with high rigidity and the heat dissipation of nanomaterials, and the theoretical and experimental research on structural defects has been of continuous interest to researchers [35,36,37]. Figure 6d shows the heat transfer network for thermal conductance measurements. First, in order to measure thermal conductance using SThM, the heated probe is brought into contact with the sample. At this time, the temperature of the probe changes due to the amount of heat transferred from the heated probe to the sample. Because the transferred heat is determined by the thermal properties of sample, the thermal conductance of the sample is locally measured by the local temperature measurement using the heated SThM probe.

In the thermal conductance measurement shown in Figure 6, the probe was heated to a temperature ~120 K higher than that of the ambient temperature (~293 K). The HOPG sample was placed in the same vacuum chamber. Figure 6a–c show the topography (a) and temperature profiles (b,c) obtained by scanning the HOPG surface using the heated probe. No specific differences in the topography are evident in Figure 6a. However, Figure 6b shows a remarkable tiny difference of the thermal conductance. The cause of the temperature difference can be understood from the schematics shown in Figure 6e,f. HOPG is composed of stacked graphene sheets, and parts that are partially cracked or torn out may exist together on the surface, as shown in Figure 6e. Because this part is an ultrathin film with a step height of less than 1 nm, there is no specific difference in the surface. However, in relation to the difference in the overlapping of these sheets, a difference in the heat transfer may occur depending on the interaction force between the graphene sheets.

The sheets of HOPG are held together by vdW interactions between the carbon atoms [38,39]. The area where the sheet is composed of strong vdW interactions is favorable for heat transfer from the probe. In contrast, the area where the sheet on the surface of HOPG is not uniform and is composed of weak vdW interactions shows relatively poor heat transfer. Therefore, in this experiment, it was possible to conclude that the cracked or torn part of the graphene sheet was measured and the difference in heat transfer due to the changed vdW interaction at the boundary of the graphene sheet was measured. These experimental results can be explained by the results of theoretical studies on the relationship between vdW interactions and heat transfer [40]. Further research is needed for a detailed analysis of this phenomenon; however, it is important to highlight the ability of the SThM probe to measure this ultra-small difference of pico-watt level heat in the temperature distribution.

## 4. Conclusions

In the present study, an SThM with pW thermal resolution was developed to measure the heat and energy transfer phenomena of atomic materials. In particular, to achieve pW thermal resolution, the probe design was mechanically and thermally optimized, and a probe with a thermal resistance calorimeter was manufactured using the MEMS process. Subsequently, the probe performance was verified through various experiments. The probe exhibited an excellent thermal resolution of ~16 pW. Additionally, it was demonstrated that a quantitative temperature distribution measurement was possible using the probe by measuring the temperature profile of a platinum line heater. Furthermore, to establish that the probe could measure the heat transfer of atomic materials, the tiny difference in the thermal conductance, which is an ideal 2D material, was measured. It was possible to observe a clear difference by measuring the thermal conductance in the area that could not be discovered in the topography, which was considered to be because of the difference in vdW interactions between the graphene sheets. In the future, the devised pW-SThM will be actively used to measure the thermal properties of various 2D materials, such as graphene, ultrathin films prepared via ALD, self-assembled monolayers, and polymer layers, and is expected to significantly contribute to the field of heat transfer measurements at the atomic scale.

## Data Availability

Not applicable.

## Supplementary Materials

The following supporting information can be downloaded at: https://www.mdpi.com/article/10.3390/nano12091479/s1, Figure S1: Input current-temperature graph of a metal line heater; Figure S2: FEM analysis of the stiffness of the pW-SThM probe; Figure S3: Mirror circuit for eliminating the influence of the drift signal; Figure S4: FEM results depicting top-view and cross-sectional view. References [26,41,42,43,44] are cited in the supplementary materials.
