# Supplementary material for: Pico-Watt Scanning Thermal Microscopy for Thermal Energy Transport Investigation in Atomic Materials
Source: Nanomaterials (Basel). 2022 Apr 27;12(9):1479. doi: 10.3390/nano12091479 (PMC9100069; doi:10.3390/nano12091479)
Supplement: Supplementary file 1 [file nanomaterials-12-01479-s001.zip › nanomaterials-1691763-supplementary.pdf]

Supporting Information

Pico-Watt Scanning Thermal Microscopy for Thermal Energy Transport Investigation in Atomic Materials

Seunghoe Koo, Jaehee Park and Kyeongtae Kim *

Department of Mechanical Engineering, Incheon National University, Incheon 22012, Korea; shkoo7736@gmail.com (S.K.); pekyk2959@gmail.com (J.P.)

* Correspondence: kyekim@inu.ac.kr

Supporting information 1. Measurement of temperature of the heater sample using the 3ω method

To determine the temperature of the metal heater used in this study, a 3ω method was employed. The results are presented in Figure S1.

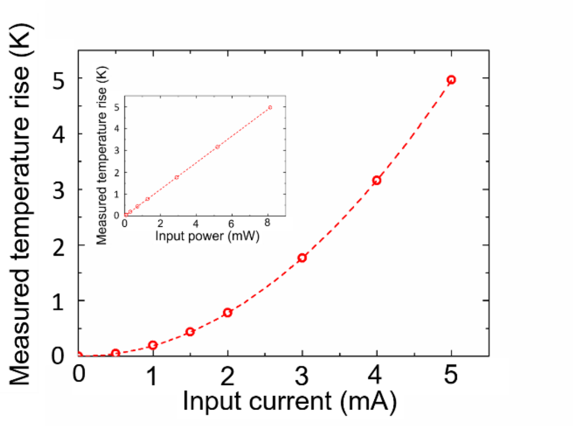

Figure S1. Input current-temperature graph of a metal line heater obtained via the 3ω method. Inset shows the temperature change according to the input power.

Supporting information 2. Spring constants of probe

The stiffness of the probe was estimated using the finite element method (FEM), as in our previous study [1]. The probe can be divided into the cantilever part, which accounts for most of the bending, and the body part where the sensor operates. The body part is larger than the cantilever, and has a groove, so that it does not bend easily and impair the mechanical stiffness. The cantilever with a U-shaped groove was fabricated from SiN_x with a length of ~200 m and a thickness of ~500 nm (Figure S2). In this calculation, Young's modulus and Poisson's ratio of silicon nitride was assumed to be 289 GPa and 0.20, respectively [2]. To estimate the stiffness of the cantilever, a force of 1 nN was applied to one end of the cantilever and the other end was fixed (Figure S2). The stiffness of the cantilever calculated from the deflection was 1.86 N/m.

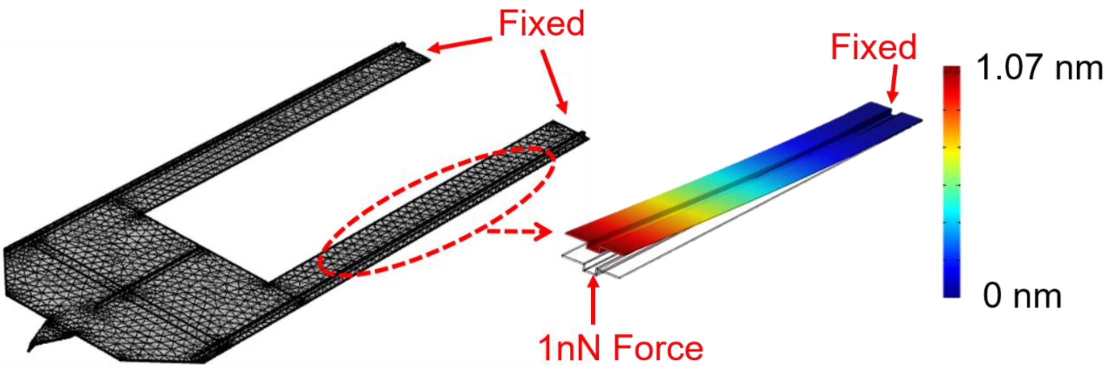

Figure S2. FEM analysis of the stiffness of the pico-watt SThM probe via meshing. Force of 1 nN was applied in the normal direction to the end of the cantilever.

Supporting information 3. Mirror circuit for eliminating temperature drift effect

A mirror circuit was developed to eliminate the effect on temperature drift noise due to ambient temperature while recording DC measurements through DC heating. The voltage across each resistor was monitored. Figure S3 shows the circuit configuration.

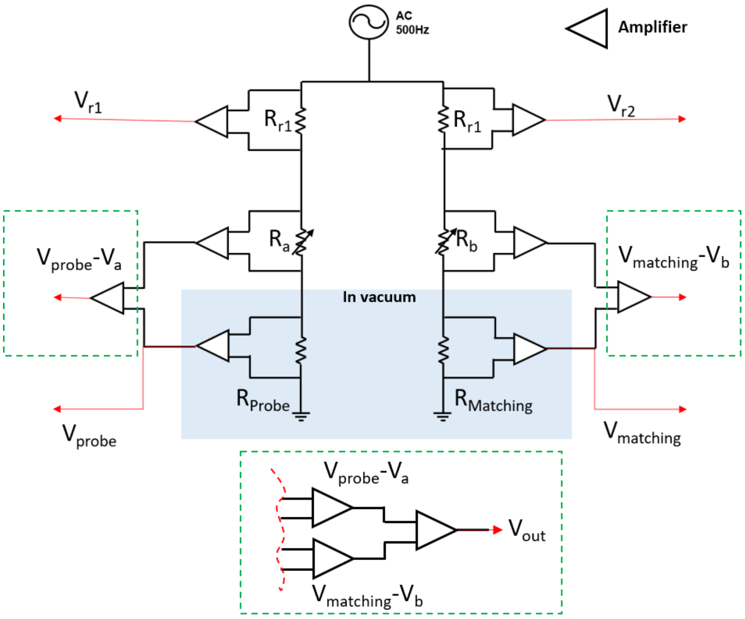

Figure S3. Mirror circuit for eliminating the influence of the drift signal. V_{r1}, V_{r2}, V_{probe}, $V_{matching}$, V_a, V_b, V_{out}, R_{r1}, R_{r2}, R_a, R_b, R_{probe}, and $R_{matching}$ are the voltage output of reference resistor 1, voltage output of reference resistor 2, voltage output of sensing probe, voltage output of matching probe, voltage output of resistor a, voltage output of resistor b, output voltage, electrical resistance of reference resistance 1, reference resistance 2, resistance a, resistance b, probe resistance, and matching probe resistance, respectively.

Supporting information 4. Finite element modeling (FEM) for evaluating the temperature distribution of the metal heater sample

For comparison with experimental results of the metal-line heater, a theoretical temperature profile was determined. Power was applied to a Pt line (width: 2.5 μm, thickness: 50 nm) fabricated on a 500 nm thick silicon dioxide film, and the temperature distribution was investigated. Since the adhesion layer is very thin, the temperature effect of adhesion layer is negligible. Figure S4 shows the simulation results for the temperature distribution. Because metal thin films have a high thermal conductivity, the surface temperature of the metal is almost the same. However, as the distance from the thin metal film gradually

increases, the temperature of the surface rapidly decreases. Here, the thermal conductivity values of Pt, SiO_2, and Si used are 77.8, 1.1, and 150 W/m K, respectively [3–5].

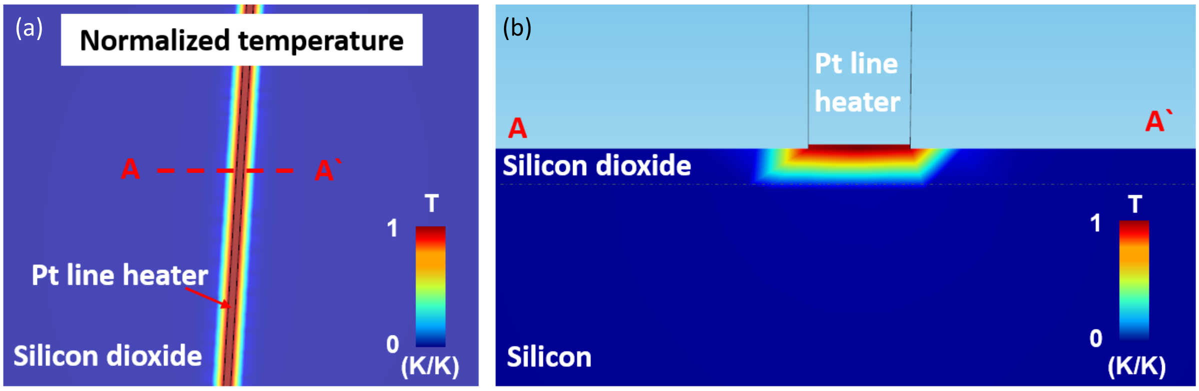

Figure S4. FEM results depicting (**a**) top-view and (**b**) cross-sectional view for the normalized temperature distribution of the metal heater sample.

References

1. Kim, K.; Jeong, W.; Lee, W.; Reddy, P. Ultra-High Vacuum Scanning Thermal Microscopy for Nanometer Resolution Quantitative Thermometry. *ACS Nano* **2012**, *6*, 4248–4257.
2. Yang, J.; Gaspar, J.; Paul, O. Fracture Properties of LPCVD Silicon Nitride and Thermally Grown Silicon Oxide Thin Films From the Load-Deflection of Long Si3N4 and SiO2/Si3N4 Diaphragms. *J. Microelectromech. Syst.* **2008**, *17*, 1120–1134.
3. Terada, B.Y.; Ohkubo, K.; Mohri, T. Thermal Conductivities of Platinum Alloys at High Temperatures. *Platinum Metals Rev.* **2005**, *49*, 21–26.
4. Kleiner, M.B.; Kuhn, S.A.; Weber, W. Thermal conductivity measurements of thin silicon dioxide films in integrated circuits. *IEEE Trans. on Electron Devices* **1996**, *43*, 1602–1609.
5. Maire, J.; Anufriev, R.; Hori, T.; Shiomi, J.; Volz, S.; Nomura, M. Thermal Conductivity Reduction in Silicon Fishbone Nanowires. *Sci. Rep.* **2018**, *8*, 4452–4459.
